# Beyond Personal Empathy: Perceiving Inclusive Empathy as Socially Shared Predicts Support for Transitional Justice Mechanisms

**DOI:** 10.1007/s42761-021-00086-2

**Published:** 2021-12-02

**Authors:** Sandra Penić, Daniel Dukes, Guy Elcheroth, Sumedha Jayakody, David Sander

**Affiliations:** 1 Swiss Centre for Affective Sciences, University of Geneva, Geneva, Switzerland; 2 University of Fribourg, Fribourg, Switzerland; 3 University of Lausanne, Lausanne, Switzerland; 4 International Centre for Ethnic Studies, Kandy, Sri Lanka

**Keywords:** Empathy, Social influence, Perceived social norms, Pluralistic ignorance, Transitional justice

## Abstract

In countries emerging from civil war, inclusive empathy is important for conflict resolution yet may be difficult to promote. Widening the predominant focus on personal inclusive empathy for conflict resolution, we examine whether support for transitional justice mechanisms (TJ) can be predicted by how much an individual perceives inclusive empathy as being shared in their local communities. Our results, based on a probability sample survey in post-war Sri Lanka (*N* = 580), reveal that the effects of this perceived communal inclusive empathy can be distinguished from those of personally experienced inclusive empathy, and that the more respondents perceive inclusive empathy as prevalent in their communities, the more they support TJ mechanisms. However, the results also indicate the contextual limits of perceived communal inclusive empathy as a resource for conflict resolution: participants tend to underestimate the prevalence of inclusive empathy, especially in militarized minority communities, and the more they underestimate it, the less they support TJ mechanisms. This study corroborates the importance of social influence in conflict resolution, suggesting that perception of inclusive empathy as shared in one’s community is a key determinant of popular support for conflict-transforming policies.

**Supplementary Information:**

The online version contains supplementary material available at 10.1007/s42761-021-00086-2.

In this paper, we examine whether inclusive empathy — i.e., empathy for one’s fellow citizens affected by diverse and complex conflict experiences regardless of their ethnic, religious, or other (politicized) group belonging — can serve as a resource for conflict transformation. Going beyond the predominant focus in the literature on personally experienced empathy for conflict resolution (see Halperin, 2016), we focus on people’s perception of the empathy of relevant others. Specifically, we examine whether the perceived communal inclusive empathy — i.e., the perception of one’s local community members’ inclusive empathy — is related to support for conflict-transforming transitional justice (TJ) policies. Building on the emerging literature on the importance of perceived social norms for conflict resolution (see Paluck, 2009; Tankard & Paluck, 2016), this study is guided by the assumption that popular support for TJ is driven not only by what people *personally* feel, but also by their perception of what *relevant others* feel. We focus on local community members (neighbors) as ‘relevant others’, typically shown as an important source of social influence for many people (see Matthes et al., 2018). As such, we contrast an individual’s *personal inclusive empathy* for all their fellow citizens (i.e., in the same country), with their *perceived communal inclusive empathy* — how much they perceive inclusive empathy to be shared among the members of their own local community.

We test the novel hypothesis that the more people perceive inclusive empathy as shared in their local communities, the more likely they are to support conflict transformation through TJ. TJ encompasses a diverse range of policies that have been implemented in the aftermath of authoritarian regimes or wars with the aim of fostering justice, reconciliation, and non-recurrence of human rights abuses (Teitel, 2002). It can include both restorative and retributive justice measures (Wenzel et al., 2008): key mechanisms typically include war crimes prosecutions, truth-telling processes, and reparations for victims (David, 2017). In postwar Sri Lanka, the present study context, such mechanisms have thus far been implemented only very partially and half-heartedly. The ideas of a truth commissions or a special war crimes tribunal remain particularly controversial and actively challenged by the war-winning state representatives and their political supporters, although a large popular consultation, conducted several years ago by a government-mandated task force, highlighted their potential contribution to constructive conflict transformation (Saravanamuttu, 2021).

## Inclusive Empathy for Diverse Conflict Experiences and Dealing with the Past

In conflict-affected societies, collective experiences of violence are typically complex and diverse: Experiences of suffering tend to co-exist with experiences of resilience and may include experiences that question the logic of the conflict, or that contradict a simple “us vs. them” perspective (Elcheroth et al., 2019). Examples include instances of helping and solidarity across frontlines (Broz, 2014), and of *intra*group violence, when people are harmed by their ingroup members (Brubaker & Laitin, 1998). To a certain degree, such experiences are likely to occur in most conflicts and affect people beyond the conflict-defined cleavage (Elcheroth et al., 2019). Previous studies suggest that learning about diverse conflict experiences may be an important factor in promoting reconciliation (Čehajić-Clancy & Bilewicz, 2017). Here, we further examine whether empathy for diverse and complex conflict experiences predicts individuals’ support for TJ mechanisms.

Empathy is a construct that is used with various meanings in the affective sciences, but here, we understand it to mean being emotionally affected by others’ suffering, experiencing compassion and sympathy for the other as a consequence (Klimecki, 2019).

More specifically, we focus on *inclusive* empathy for one’s co-citizens affected by diverse and complex conflict experiences, irrespective of their background. It is widely assumed that inclusive empathy promotes conflict resolution: experiencing empathy for the “other” can motivate endorsement of conflict-transforming beliefs and behaviors (see Halperin, 2016). Indeed, some previous studies have shown that, in the context of intergroup conflicts, feeling empathy for outgroup members can foster positive outgroup attitudes (Dovidio et al., 2009), decrease support for aggressive policies (Kaukiainen et al., 1999), and increase support for restorative policies (Brown & Cehajic, 2008). Yet, previous studies also show the limits of personal inclusive empathy in promoting conflict resolution: such empathy may be scarce in conflict-settings because people typically experience less empathy for outgroup members (Dovidio et al., 2009; Leach et al., 2003). Even when they experience it, some studies show that empathy for the other does not necessarily motivate the endorsement of conflict-transforming beliefs or behaviors (see Dixon et al., 2012; Rosler et al., 2017). Accordingly, personal inclusive empathy alone may be insufficient to promote conflict resolution (Halperin, 2016; Zaki & Cikara, 2015).

Conflict-transforming beliefs and behaviors, however, are not only influenced by personal emotions. People do not live in a vacuum but are part of broader groups and are deeply affected by social norms — i.e., beliefs, attitudes, and customs, perceived as prevalent or desirable in a group (Miller & Prentice, 1996). In the field of conflict resolution, a growing number of studies emphasize the importance of social influence, indicating that it may be particularly fruitful to target the perception of social norms in order to decrease destructive behaviors and promote conflict resolution (Paluck, 2009; Tankard & Paluck, 2016). Whereas most of these previous studies have focused on people’s perception of others’ beliefs and behaviors, the role of emotional social influence for conflict resolution has thus far been less studied.

## Beyond Personal Empathy: The Role of Perceived Communal Empathy

Previous studies show that the perception of relevant others’ emotions can shape people’s personal emotions, as well as their beliefs and behaviors (e.g., Conejero & Etxebarria, 2007; Kaakinen et al., 2021; Kim, 2016). Focusing on empathy, two examples from the literature highlight the role that perceived empathy within a group can play. Firstly, Nook et al. (2016) showed that when group members perceive empathy for stigmatized social targets as prevalent (i.e., as normative) in their groups, they are more likely to experience it and to act prosocially toward those targets (Nook et al., 2016). Similarly, in an intergroup context, highlighting empathy for outgroup members as prevalent in one’s group can enhance personal empathy and more positive outgroup attitudes among the group members (Tarrant et al., 2009).

Whether the perception of empathy as normative may similarly serve as a resource for conflict resolution has largely remained understudied. In the context of the Israeli-Palestinian conflict, Nadler and Liviatan (2006) have shown that exposing Israeli participants to the expressions of empathy for Israelis from an *outgroup* member (i.e., a Palestinian political leader) increases their willingness to reconcile. The main novelty in the present study is the focus on the perceived prevalence of inclusive empathy among *ingroup* members — i.e., among one’s local community members (neighbors). More specifically, we examine the role of this *perceived communal inclusive empathy* — conceptualized as the perception of prevalence of inclusive empathy in one’s community (i.e., among one’s neighbors) — in predicting support for TJ. We hypothesize that when people perceive inclusive empathy as collectively shared in their community, they will be more likely to support TJ policies that concern them collectively. People are especially motivated to understand and follow the norms of social groups to which they belong and care about (Tankard & Paluck, 2016). Hence, we focus on one’s local community, as one of the most influential social groups for many people (McNamara et al., 2021; Stevenson et al., 2021). For example, recent meta-analyses show that people are particularly influenced by their neighbors (as opposed to strangers, politicians, or the media), on political issues that are of direct relevance for their (shared) daily lives (see Matthes et al., 2018). Please note that as both are examples of inclusive empathy, we will henceforth use the terms *personal empathy* and *perceived communal empathy*, without including the word “inclusive” each time.

## Self-expressions and Collective Perceptions: the Possible Impact of Perceiving Personal Empathy as Exceptional

There are good reasons to expect that perceptions of inclusive empathy as collectively shared do not easily reflect aggregate tendencies of personal empathy. On one hand, to the extent that empathy might be valued positively, people could be motivated to attribute more of this quality to themselves than to others. Similar so-called better-than-average effects have been documented in various domains, including emotions (e.g., Goldenberg et al., 2014; Ong et al., 2018). On the other hand, people have less direct information concerning the inner states of others compared to themselves and, therefore, have to rely more on indirect cues, which could be manipulated and/or prone to different forms of (self-) censorship, when it comes to forming perceptions of social norms (Tankard & Paluck, 2016). When there are systematic discrepancies between private beliefs and public behavior, people can be led to wrongly believe that others’ private beliefs are different to their own, a phenomenon dubbed *pluralistic ignorance* since Prentice and Miller’s (1996) classic work. Some recent studies have also documented the phenomenon of pluralistic ignorance for emotions: for example, Jordan et al. (2011) have shown that people tend to underestimate the prevalence of others’ negative emotions, because negative emotions are more often suppressed in social settings.

One major source of the perception of others’ emotions is the observation of emotional expressions, such as in social interactions, public events, and collective gatherings (Manstead & Fischer, 2001; Mumenthaler & Sander, 2012, 2019). In post-conflict societies, emotions about past conflict are often publicly expressed through the commemoration of collective losses or victories (Rimé et al., 2010). These tend to be limited however to the expression of official narratives of the victor and to be less open to critical and diverse conflict memories (Jessee, 2017; McDowell & Braniff, 2014). Moreover, in asymmetric conflicts, the powerful side may aim to suppress the voices and memories of entire groups of people (McDowell & Braniff, 2014). In heavily militarized and surveilled minority communities, opportunities for public expression of empathy with minority experiences may be particularly limited (e.g., de Mel, 2007). Consequently, lower support for TJ may be driven not (only) by a lack of personal empathy but also, and possibly more critically, by the lack of a clear perception that empathy is shared by other community members. Clear enough perceptions might be particularly difficult to reach on politically sensitive issues, such as empathy with experiences that belie dominant conflict narratives, and in contexts where expressing empathy publicly can be risky, such as in heavily surveilled minority communities.

## Summary and Hypotheses

To summarize, we examine the role of inclusive empathy for diverse conflict experiences — beyond simple us vs. them conflict narratives — in predicting people’s support for three key TJ mechanisms, which have been considered and publicly debated in the present study context, but never wholeheartedly embraced by powerholders, nor systematically implemented: truth-telling, prosecutions, and reparations. Building on previous studies that stress the role of social influence in conflict resolution, we examine the impact of perceived communal empathy on support for TJ. We hypothesize that the more individuals perceive their community members as empathetic, the more they will support TJ. Yet, the perception of communal empathy may be a hard-to-reach resource for conflict transformation: In communities emerging from collective violence, opportunities for public expression of inclusive empathy may be limited, making it difficult to know to what extent empathy is shared by other community members. This might create or exacerbate a tendency for people to assume that their own capacity to be empathetic is exceptional in their community. We expect such perceptions of personal empathy as being exceptional to play a detrimental role: the more people tend to perceive a gap between own and others’ levels of inclusive empathy, we hypothesize, the less they will support potentially conflict-transformative but politically controversial TJ policies.

## Study Context

## Method

Post-war Sri Lanka is currently reviving from a civil war that lasted almost three decades. The conflict broke out between the Sri Lankan state forces, dominated largely by the country’s Sinhalese majority (comprising 74.9% of the population), and an armed militant group, the Liberation Tigers of Tamil Eelam (LTTE), which claimed to represent the aspirations of the largest minority ethnic group, ethnic Tamils (comprising 15.3% of the population; Census of Population and Housing 2012, 2015). Most of the fighting took place in the Northern and Eastern provinces, predominately inhabited by the Tamil and Muslim minorities. The civil war ended brutally in 2009 with military victory for the government and the annihilation of the LTTE. The Sri Lankan government subsequently implemented a unilateral peace process that prioritized infrastructure development and economic recovery, while side-lining issues of reconciliation and justice, despite international pressure (see “OHCHR Sri Lanka”, 2018) and popular demands for truth-seeking, accountability, and reparations, especially among the country’s ethnic minorities (see “Final report CTF”, 2017).

The plight of the minorities, especially of Tamils in the war-torn North and East, has been dire. Since the cessation of the war, civilian lives in these areas have been affected by a high military presence, constant surveillance, intimidation, and a culture of impunity in relation to abductions, enforced disappearances, extrajudicial killings, and rape (Amnesty International, 2017; Human Rights Watch, 2020). Monuments and cemeteries of the fallen LTTE that once stood in the North have been demolished and replaced in some cases by military infrastructure, or by statues and memorials celebrating the state’s military victory (Haviland, 2011; Hyndman & Amarasingam, 2014; Tamil Guardian, 2016). Commemorations and communication activities in relation to conflict experiences have been strongly curtailed for the Tamil people in these areas. For example, the May 18th commemorations in Mullivaikal, which remembers the colossal loss of civilian life during the final phase of the war, has been suspended through court orders, while victory day parades in the South have been carried out in grandeur and through state patronage (Groundviews, 2017).

The living conditions of the war-affected Sinhalese communities in North Central Sri Lanka are also difficult. (These communities are popularly referred to by the media and relief organizations as “border villages” because of their location near former frontlines and territory controlled by the LTTE during the civil war). During the war, these communities received very little state/NGO assistance while facing the brunt of violence. Amidst both heavy military and LTTE presence, these communities possessed hybrid identities, and often maintained cordial socioeconomic relations with Tamil and Muslim communities abreast and across the “border.” Despite the fact the war has ended, they continue to experience a heavy military presence and seem excluded from Sri Lanka’s post-war development. Living in extreme poverty and being remote from central institutional support structures, they also seem excluded from the collective memory of the conflict hegemonized by the state (de Silva et al., 2019; Korf & Silva, 2003; Rajasingham-Senanayake, 2011).

### Sample

The data stem from a survey conducted within the framework of an international research project in September and October 2019, among probability samples from two communities affected by war violence in Sri Lanka: among Tamils from the district of Kilinochchi, in the Northern province, where they form the local majority (hereafter the “Northern Tamils”), and among Sinhalese from the district of Anurhadhapura, in the North Central province (i.e., “Sinhalese border villages,” see above). In each region, 20 local communities (i.e., *Grama Niladhari* division, corresponding to the village level) were first randomly selected, and then 15 target respondents were randomly selected from the adult population (i.e., registered voters) within each community (the final sample size per community varies from 8 to 15, with a mean sample size of 14.50). Informed consent was obtained from all research participants. They were interviewed in their language by trained interviewers from the broader region. The final sample consists of 580 respondents (280 from the Sinhalese border villages and 300 from the Northern Tamils).

### Measures

Personal empathy for one’s co-citizens affected by diverse conflict experiences, irrespective of their background (i.e., inclusive empathy), was assessed with the use of vignettes (Alexander & Becker, 1978). Respondents read seven vignettes that described diverse conflict events: two vignettes described experiences of victimhood (i.e., loss of life and affected livelihood); two vignettes described instances of resistance (i.e., community coordination to resist enemy attacks) and resilience (i.e., helping a community member affected by war violence); and three vignettes described events that challenge the representation of us vs. them: one vignette described malleability of group boundaries/relations (neighbors turning against each other), another described solidarity across group boundaries (being helped by an outgroup member), and the third described an instance of witnessing and condemning ingroup perpetration. All vignettes were based on extracts from interviews with witnesses of collective violence in Sri Lanka collected within the scope of an international research project. To access diverse conflict experiences, particular efforts were devoted to reaching participants who differ in terms of their politicized markers of identity, generation, gender, political involvement, and educational, economic, and social backgrounds. The extracts were transformed to vignettes by summarizing and simplifying the description of the main event, and by removing information about the identity and ethnicity of the narrator. The resulting vignettes were presented to participants as featuring real people and being based on true events from the Sri Lankan conflict.

The following vignette described an instance of intergroup solidarity:

My neighbor opened her home to hide us when some people in our neighborhood went on a rampage destroying property and killing people. Despite belonging to a different ethnic group than us, she housed my family and me for some days. Then someone had come and threatened our neighbor that if they harbor people of our ethnicity, their home too will be burned. My neighbor said it was getting very dangerous. But she continued to hide us upstairs in her home.

After each of the seven vignettes, respondents were asked to rate the item “I empathize with (feel for) people who experienced such an event” on a 1 (strongly disagree) to 6 (strongly agree) scale. *Personal empathy* for diverse experiences was operationalized as a mean of seven items (the above item per seven vignettes). Principal component analysis showed that all items load on one factor (which explains 57.4 % of variance, with factor loadings ranging from .70 to .83), and the internal reliability is high (Cronbach’s alpha is .87).

To assess perceived communal empathy, after each vignette, respondents were invited to imagine that one of their neighbors experienced a similar event. They were then asked: “If he/she told other neighbors about this event, how likely is that they [other neighbors] would express support or empathy for him/her?” on a 1 (Very unlikely) to 6 (Very likely) scale. *Perceived communal empathy* was operationalized as the mean across 7 items (the above item per each of seven vignettes). Principal component analysis showed a one-factorial structure (explaining 72.34 % of variance, with factor loadings ranging from .83 to .91), and high internal reliability (Cronbach’s alpha is .93). As such, the diverse experiences expressed in the vignettes could be used to compare *personal empathy* for an event — “how I feel” — with *perceived communal empathy* — “how I think my neighbors feel” — about the same event.

Support for transitional justice was assessed with three measures:

*Support for truth commissions* was assessed with two items. Respondents were asked to what extent a truth commission in Sri Lanka would be helpful or harmful “to promote justice”, and “to reconcile communities”, rated on a 1 (very harmful) to 5 (very helpful) scale. The composite indicator was computed as a mean of the two items, showing high internal reliability (Cronbach’s alpha is .89).

*Support for prosecutions* was similarly assessed with two items, asking respondents to what extent criminal prosecutions of war crimes would be helpful or harmful to promote justice, and to reconcile communities, rated on a 1 (very harmful) to 5 (very helpful) scale. The composite indicator was computed as a mean of the two items, showing high internal reliability (Cronbach’s alpha is .92).

*Support for reparations* was measured with four items: “The government should provide reparations to members of all ethnic groups who were harmed during the war and not just to my group”; “Economic development by the state should benefit all ethnic groups in all regions, instead of focusing on only some ethnic groups in certain regions of the country”; “Land that was dispossessed during the conflict should be compensated by the state to the legal owners”; and “The government should help refugees and displaced persons to repossess their properties and return to their homes,” rated on a 1 (strongly disagree) to 6 (strongly agree) scale. The composite indicator was computed as a mean of the four items, showing good internal reliability (Cronbach’s alpha is .71).

### Control variables

We controlled for personal exposure to conflict-related events (*conflict victimization)*, operationalized as the number of events experienced among the following: being expelled, being imprisoned, being wounded, experiencing the death or disappearance of a family member, suffering property loss or damage, or being looted (total score ranging from 0 to 8).

We further controlled for classic sociodemographic variables: sex, age, and level of education (whether the respondent has finished the secondary level or higher). We further controlled for the survey location (i.e., Northern Tamils vs. Sinhalese border villages).

Descriptive statistics for all variables are shown in Table 1.

**Table 1:** Descriptives

	Number	% of the sample	Min	Max	Mean	SD
Female	580	61%				
Secondary or higher education	560	51%				
Sinhalese	580	48%				
Tamils	580	52%				
Age	580		19	88	44.76	16.41
War victimization	579		0	8	1.99	2.07
Personal empathy	580		2.29	6	5.07	0.65
Perceived communal empathy	577		1	6	4.45	1.05
Support for truth commissions	540		1	5	3.98	0.80
Support for prosecutions	554		1	5	3.90	0.88
Support for reparations	579		3.25	6	5.23	0.52

## Results

The more people perceive inclusive empathy for diverse conflict experiences as prevalent in their communities, the more they personally empathize with these experiences (*r* = .603, *N* = 507, *p* < .001). We next examined how both personal and perceived communal empathy are related to participants’ support for three different transitional justice mechanisms. To this end, we performed linear regression analyses with IBM SPSS Statistics software (Version 25). Due to the large sample size and small percentage of missing data on outcome variables (0.2 % for support for reparations, 4.5 % for support for truth commissions and 6.9 % for support for prosecutions), cases with missing values were deleted listwise (Allison, 2002).

In a first step, we regressed personal empathy on three measures of support for transitional justice, while controlling for socio-demographics, war victimization, and the survey location (i.e., Northern Tamils vs. Sinhalese border villages). The results are shown in Table 2 (Model 1).

**Table 2:** Linear regression models of the impact of personal and perceived communal empathy on support for transitional justice mechanisms

	Support for truth commissions			Support for prosecutions			Support for reparations		
	B (SE)	95% CI	Beta	B (SE)	95% CI	Beta	B (SE)	95% CI	Beta
*Model 1*									
Constant	2.761*** (.301)	2.169, 3.352		2.294*** (.324)	1.657, 2.931		3.931*** (.201)	3.536, 4.326	
Female	.103 (.066)	− .026, .233	.064	.147* (.070)	.008, .285	.081	.045 (.043)	− .040, .130	.042
Age	.004 (.002)	.000, .009	.092	.004 (.002)	.000, .009	.082	.000 (.001)	− .003, .003	− .004
Secondary or higher education	.025 (.071)	− .115, .165	.016	− .028 (.076)	− .178, .122	− .016	− .002 (.047)	− .095, .090	− .002
Northern Tamil	.811*** (.097)	.620, 1.002	.510	1.028*** (.104)	.824, 1.231	.581	.175** (.064)	.049, .301	.167
War victimization	− .034 (.022)	− .077, .008	− .091	− .050* (.023)	− .096, − .004	− .119	.056*** (.015)	.028, .085	.222
Personal empathy	.115* (.054)	.009, .222	.095	.175** (.058)	.060, .290	.129	.212*** (.036)	.141, .283	.263
Model fit	Adjusted R-square = .175, *F*(6/513) = 19.376, *p* < .001			Adjusted R-square = .219, *F*(6/527) = 25.918, *p* < .001			Adjusted R-square = 0.125, *F*(6/551) = 14.268, *p* < .001		
*Model 2*^*1*^									
Personal empathy	− .044 (.061)	− .163, .076	− .036	.071 (.066)	− .059, .201	.052	.156*** (.041)	.075, .236	.193
Perceived communal empathy	.224*** (.042)	.141, .306	.298	.148** (.045)	.059, .237	.177	.081** (.028)	.025, .136	.161
	R-square change = .043, *F* change(1/512) = 28.324, *p* < .001			R-square change = .015, *F* change(1/526) = 10.703, *p* < .001			R-square change = .013, *F* change(1/550) = 8.190, *p* = .004		
Model fit	Adjusted R-square = .217, *F*(7/512) = 21.539, *p* < .001			Adjusted R-square = .233, *F*(7/526) = 24.154, *p* < .001			Adjusted R-square = 0.136, *F*(7/550) = 13.559, *p* < .001		

^1^Model 2 controlling for same variables as Model 1

*p < .05; **p < .01; ***p < .001

In line with our expectations and previous studies, we found that the more participants reported feeling personal empathy for fellow citizens affected by diverse conflict experiences, the more they support truth commissions, prosecutions, and reparations. In the next step, we added *perceived communal empathy* to the models (see Table 2, Model 2). Perceived communal empathy explained an additional 4.3% of variance for support for truth commission, 1.5% of support for prosecutions, and 1.3 % of support for reparations (without controlling for personal empathy, perceived communal empathy explains 4.9%, 2.7%, and 4.5% of variance of support for truth commissions, prosecutions, and reparations, respectively). In line with our expectations and theoretical model, we find that the more participants perceive inclusive empathy as socially shared in their communities, the more they support the three TJ mechanisms. The impact of the perceived communal empathy is statistically significant for all three outcomes, even when controlling for personal empathy. Moreover, after introducing the measure of perceived communal empathy, the impact of personal empathy ceases to be statistically significant for support for the two most controversial TJ mechanisms (prosecutions and truth-telling).

Overall, our findings indicate that support for transitional justice is not only shaped by personal empathy, but rather, it is also shaped by the perception that inclusive empathy is shared in one’s community: indeed, the perceived communal empathy predicts respondents’ support for TJ mechanisms over and above the effect of their personal empathy.

We performed the following additional analyses to check the robustness of our findings (see Supplementary material for more detail).

First, we examined whether the results depended on the type of conflict experiences. On average, respondents showed slightly more personal and perceived communal empathy for narratives of collective victimhood (i.e., vignettes 1 and 2) than for more complex experiences (i.e., vignettes on blurred boundaries, see Supplementary material, Table S1). We performed the same regression analyses as shown in Table 2 (Model 2), but separately with measures of personal empathy and perceived communal empathy, for each of the seven vignettes (i.e., instead of the composite indicator across all vignettes). We found similar results (to those described above) for all individual vignettes as for the composite measure presented here (see Supplementary material, Table S2).

Second, we performed additional analyses to rule out alternative explanations of our findings. More specifically, support for TJ could perhaps be driven by aspects of collective memory — such as the knowledge about or the perceived local prevalence of diverse conflict experiences (e.g., Čehajić-Clancy & Bilewicz, 2017) — rather than by inclusive *empathy* for these experiences. We performed the same regression analyses as shown in Table 2 (Model 2), while controlling for the respondents’ knowledge about diverse conflict experiences and their perceived prevalence of these experiences in their local communities (Supplementary material, Table S3). Perceived communal empathy remained a statistically significant predictor in all models suggesting that it retained some explanatory power.

### Role of the Gap Between Personal and Perceived Communal Empathy

Having established that perceived communal empathy matters for support for TJ, as a next step, we examined whether respondents tended to perceive their community members as having less inclusive empathy than themselves, and whether doing so has implications for their support for TJ mechanisms.

First, in line with our hypothesis, we found that respondents tended to perceive their community members’ inclusive empathy as lower than their own (personal empathy *M* = 5.075, SD = 0.650; perceived communal empathy *M* = 4.450, SD= 1.048; paired samples *t* test: *t* = 17.969, *df* = 576, *p* < .001).

**Fig. 1:**
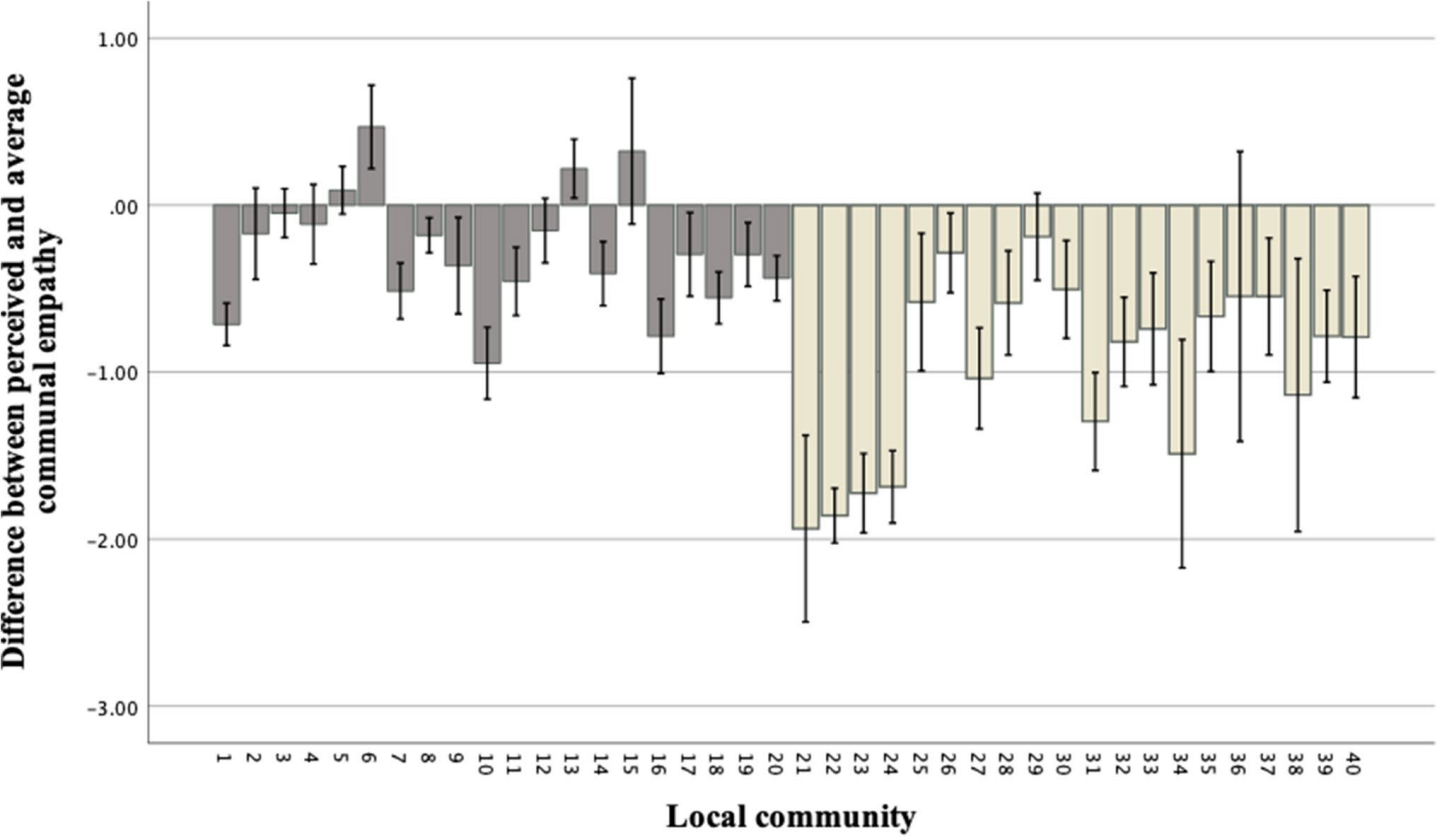
Difference between the respondents’ perceived communal empathy and the actual average personal empathy in their communities with 95% confidence intervals, in Sinhalese (dark grey) and Northern Tamil (light grey) communities

We further examined the differences between respondents’ estimates of their neighbors’ inclusive empathy (i.e., perceived communal empathy) and the average of personal empathy reported by their fellow villagers, across 40 local communities. As Figure 1 illustrates, in many communities, average perceptions of communal empathy were lower than were average expressions of personal empathy: in some communities, the gap was as much as of almost 2 points on a 6-point scale.

Second, we examined whether the gap between perceived communal and personal empathy (individual or aggregate) predicted respondents’ support for TJ mechanisms. More specifically, we computed the indicators of (1) the difference between personal and perceived communal empathy, and (2) in each community, the difference between the average level of personal empathy in the community and perceived communal empathy among the community inhabitants and regressed them on support for the three TJ mechanisms.

As predicted, we found that the more respondents perceived their neighbors’ inclusive empathy as lower than their own, the less support they showed for truth commissions and prosecutions. There was no significant relation with support for reparations (see Table 3, Model 1). Similarly, the more the participants estimated their neighbors’ inclusive empathy as lower than the average inclusive empathy in the local sample, the less support they expressed for truth commissions and prosecutions (Table 3, Model 2).

**Table 3:** Linear regression models of the impact of the difference between personal and perceived communal empathy (Model 1) and average communal and perceived communal empathy (Model 2) on support for transitional justice mechanisms

	Support for truth commissions			Support for prosecutions			Support for reparations		
	B (SE)	95 % CI	Beta	B (SE)	95 % CI	Beta	B (SE)	95 % CI	Beta
*Model 1*									
Difference personal and perceived communal empathy	− .192*** (.041)	− .274, − .111	− .205	− .111* (.045)	− .198, − .023	− .106	− .039 (.028)	− .095, .017	− .062
Model fit	Adjusted R-square = .202, *F*(6/513) = 22.838, *p* < .001			Adjusted R-square = .215, *F*(6/527) = 25.313, *p* < .001			Adjusted R-square = .073, *F*(6/551) = 8.304, *p* < .001		
*Model 2*									
Difference average communal and perceived communal empathy	− .208*** (.042)	− .291, − .125	− .217	− .118* (.046)	− .207, − .028	− .110	− .041 (.029)	− .098, .016	− .064
Model fit	Adjusted R-square = .206, *F*(6/513) = 23.382, *p* < .001			Adjusted R-square = .216, *F*(6/527) = 25.418, *p* < .001			Adjusted R-square = .073, *F*(6/551) = 8.334, *p* < .001		

Models controlling for the same variables as in Table 2

*p < .05; **p < .01; ***p < .001

Overall, these findings indicate that the more the respondents perceive their personal level of empathy as exceptional within their community, the less they support the two most controversial TJ mechanisms.

Finally, we examined the relationship between the tendency to perceive one’s own empathy as exceptional and the contextual circumstances, as there are likely to be limited opportunities to freely communicate and display empathy in public in more heavily surveilled and militarized minority communities. We found that fear of surveillance of war-related communication is highly prevalent in the Northern Tamil communities: on average, about 80% of the respondents agree or strongly agree with the *statements “You can never be careful enough when talking about the war”* (on 1–6 scale, Mean = 5.21, SD = .895) and “When it comes to talking about the war, even the walls have ears” (Mean = 5.25, SD = 1.008), compared to about 50% of the Sinhalese respondents from the border villages (Mean = 4.11, SD = 1.313; *t* test: *t* = 11.802, *df* = 566, *p* < .001 and Mean = 4.14, SD = 1.339; *t* test; *t* = 11.199, *df* = 566, *p* < .001, correspondingly).

As expected, we found that the tendency to perceive communal empathy as lower than one’s own was more pronounced among the Northern Tamil respondents (where fears were greater) than among the Sinhalese respondents. (Difference personal and perceived communal empathy: Tamils: *M* = .963, SD = .907; Sinhalese: *M* = .266, SD = .516; *t* test; *t* = 11.249, *df* = 575, *p* < .001). Similarly, we found that, on average, the tendency to underestimate communal empathy compared to the average personal empathy was more pronounced in more heavily militarized minority Tamil communities than in the Sinhalese communities (see Figure 1; difference average and perceived communal empathy: Tamils: *M* = .963, SD = .907; Sinhalese: *M* = .266, SD = .516; *t* test; *t* = 11.249, *df* = 575, *p* < .001).

Furthermore, we found that the more the respondents feared the surveillance of war-related communication, the more they perceived their own empathy as exceptional (correlations with the agreement with the statements: “You can never be careful enough when talking about the war”: *r* = .256, *p* < .001 with the difference between personal and perceived communal empathy, *r* = .214, *p* < .001 with the difference between average and perceived communal empathy; and “When it comes to talking about the war, even the walls have ears” (*r* = .241, *p* < .001 with the difference between personal and perceived communal empathy; *r* = .195, *p* < .001 with the difference between average and perceived communal empathy).

Taken together, our findings suggest that people may underestimate the prevalence of inclusive empathy for diverse conflict experiences in their communities, and that this state of emotional pluralistic ignorance has detrimental implications for their support for TJ. Moreover, they suggest that the state of emotional pluralistic ignorance may be more likely when there are limited opportunities to freely communicate about the war.

## Discussion

We examined whether the perception of inclusive empathy for diverse conflict experiences as shared in one’s community predicts personal support for TJ. Two key findings emerged.

First, we found that the more people perceived empathy as shared in their local communities, the more they personally empathized with these experiences, and the more they supported the three TJ mechanisms studied here: prosecutions, truth commissions, and reparations for victims. Notably, their perception of communal empathy explained their policy attitudes over and above the effect of their personal empathy: the impact of perceived communal empathy was stronger and more consistent than that of personal empathy. This is in line with a growing number of studies that highlight the role played by social influence on conflict resolution (see Tankard & Paluck, 2016). Whereas these previous studies have focused on the perception of others’ beliefs and behaviors, the present study highlights the importance of the perception of prevalence of inclusive empathy among relevant others and corroborates previous laboratory findings on the relevance of perceived empathy norms for prosocial outcomes (Nook et al., 2016; Tarrant et al., 2009) in a real-world, post-war setting.

Second, however, while the perception of inclusive empathy as socially shared is a potential resource for conflict transformation, our findings also show that it is a fragile resource, dependent on contextual circumstances. We found that respondents tended to perceive their personal empathy as exceptional in their communities and that the more they did so, the less they supported the two most controversial TJ mechanisms (prosecutions and truth-telling). It is possible that multiple factors concur to produce the observed gap between people’s expressions of empathy and their perceptions of the likelihood of empathy among other community members. Respondents might underestimate the level of empathy among others because they lack publicly visible cues (i.e., display emotional pluralistic ignorance) and/or downplay it relatively to their own level of empathy to make themselves look better (i.e., perform a “better-than-average” effect). However, the gap did not appear invariably across the study contexts but is particularly pronounced among the more heavily surveilled minority communities and among individuals with a heightened awareness of surveillance. These contextual variations are consistent with the interpretation that a (perceived) climate of surveillance limits the opportunities for public expressions of inclusive empathy and supports the hypothesis of emotional pluralistic ignorance as at least one factor contributing to the gap. Future studies might go further in disentangling its role from that of other possible factors. The critical contribution of the present study however lies in demonstrating that the gap has plausible consequences: the more people perceive their own empathy as exceptional in the wider community, the less they tend to be supportive of conflict-transforming TJ policies. This suggests that efforts to boost people’s empathy with diverse war experiences can be ineffective, and even potentially counter-productive, if doing so only increases people’s sense of being exceptional, rather than their perception that empathy is a shared resource within their community.

The results reported in this study were robust to various controls and are based on survey data from a relatively large probability sample of respondents. Nevertheless, we acknowledge two principal limitations of our study. First, the nature of the data does not allow the drawing of causal conclusions. Future field studies should test experimentally whether removing obstacles to the perception that inclusive empathy is socially shared can foster support for TJ. Second, to clarify and disentangle the role of emotional pluralistic ignorance, future studies are needed with samples representative of the full reference group of “neighbors” and equivalent rating scales across personal and perceived communal empathy measures. Our estimates of the local average inclusive empathy were based on small local samples (average *N* = 14.5) from one ethnic community (i.e., only Tamil respondents from the Northern province, and only Sinhalese respondents from the North Central province). Moreover, whereas participants rated both personal and perceived communal empathy on a 1 to 6 rating scale, the underlying value labels were different (i.e., agreement vs. perceived likelihood). The present finding of meaningful and theoretically expected correlates with our measures of difference between personal and perceived communal empathy (i.e., stronger gap in the minority communities and negative correlations with support for TJ), which run against explanations of the observed gap in the sole terms of general psychological motives such as the “better-than-average” effect, makes a case for testing the contribution of emotional pluralistic ignorance with a specifically tailored methodology in future studies.

In sum, this study highlights the relevance of emotional social influence processes for psychosocial dynamics in conflict-ridden societies. It shows that perceived communal empathy predicts increased support for three different TJ mechanisms over and above personal empathy, and in a societal context where these mechanisms are both highly controversial and potential levers for constructive conflict transformation. It therefore suggests that it may be beneficial to extend the predominant focus on personal empathy in studies on conflict resolution (Halperin, 2016; Zaki & Cikara, 2015). When it comes to popular support for conflict-transforming policies that concern people collectively, how *we* feel may prove to be even more important than how *I* feel. Future studies in different conflict-affected societies should further investigate the potential power of socially shared inclusive empathy for conflict resolution, and the psychological, social, and political factors that promote or undermine it. Indeed, a troubling corollary of our findings is that the perception of inclusive empathy as socially rare undermines support for TJ.

Finally, our findings have implications for the rapidly growing field of emotion-related conflict resolution interventions. Whereas these interventions are typically focused on cultivating personal empathy (for discussion, see Halperin, 2016), our study suggests that to promote conflict-transformative policies, it may be particularly fruitful to aim to strengthen compassionate and inclusive norms (Zaki & Cikara, 2015). Moreover, in communities characterized by pluralistic ignorance, interventions should aim to facilitate a realistic perception of *collective capacity* for inclusive empathy (Prentice & Paluck, 2020). For example, opening public spaces for compassionate sharing of diverse conflict narratives that go beyond the simplified us vs. them perspective — such as about acts of solidarity and kindness across the divides — could strengthen the perception that inclusive empathy is shared and valued by relevant others, thereby empowering compassionate voices and their support for measures designed to bring lasting peace to post-war communities.

## Supplementary Information

ESM 1 (DOCX 31 kb)

## Additional information

### Data Availability

Data access can be requested through FORSbase at: https://forsbase.unil.ch/project/study-public-overview/16859/2/.

SPSS syntax used to conduct the analyses is available in the Open Science Framework repository: https://osf.io/ah7r6/?view_only=5883cef06fda43a094e77d3dbfb5b431.
