# Supplementary material for: Beyond Personal Empathy: Perceiving Inclusive Empathy as Socially Shared Predicts Support for Transitional Justice Mechanisms
Source: Affect Sci. 2021 Dec 2;2(4):402–13. doi: 10.1007/s42761-021-00086-2 (PMC9382919; doi:10.1007/s42761-021-00086-2)
Supplement: Supplementary file 1 — (DOCX 31 kb) [file 42761_2021_86_MOESM1_ESM.docx]

|  | Personal empathy | Perceived communal empathy |
| --- | --- | --- |
| *V1: Trapped by violence* |  |  |
| Northern Tamils | 4.99 (0.944) | 3.89 (1.302) |
| Sinhalese border villages | 5.49 (0.522) | 5.21 (0.658) |
| Total | 5.24 (0.807) | 4.53 (1.231) |
| *V2: Boundaries disrupting normal life* |  |  |
| Northern Tamils | 5.2 (0.847) | 4.1 (1.316) |
| Sinhalese border villages | 5.44 (0.497) | 5.2 (0.586) |
| Total | 5.31 (0.709) | 4.64 (1.166) |
| *V3: Daring to resist* |  |  |
| Northern Tamils | 5.07 (0.905) | 4.13 (1.358) |
| Sinhalese border villages | 5.45 (0.506) | 5.15 (0.672) |
| Total | 5.26 (0.762) | 4.62 (1.196) |
| *V4: Sticking together to survive* |  |  |
| Northern Tamils | 4.78 (0.988) | 3.88 (1.337) |
| Sinhalese border villages | 5.36 (0.51) | 5.08 (0.657) |
| Total | 5.07 (0.842) | 4.47 (1.216) |
| *V5: Friend or foe?* |  |  |
| Northern Tamils | 4.49 (1.293) | 3.47 (1.43) |
| Sinhalese border villages | 5.29 (0.569) | 4.99 (0.725) |
| Total | 4.89 (1.081) | 4.22 (1.37) |
| *V6: Support across frontlines* |  |  |
| Northern Tamils | 4.32 (1.254) | 3.33 (1.462) |
| Sinhalese border villages | 5.21 (0.568) | 4.98 (0.697) |
| Total | 4.76 (1.071) | 4.16 (1.411) |
| *V7: Not in my name* |  |  |
| Northern Tamils | 4.82 (0.926) | 4.06 (1.118) |
| Sinhalese border villages | 5.26 (0.488) | 5.06 (0.62) |
| Total | 5.04 (0.775) | 4.55 (1.039) |

*Supplementary Information*

*Beyond personal empathy: Perceiving inclusive empathy as socially shared predicts support for transitional justice mechanisms*

Table S1. Descriptives by the type of conflict narrative.

Table S2. Linear regression models of the impact of personal and perceived communal empathy for separate conflict narratives on support for transitional justice mechanisms.

|  | | Support for truth commissions | | | | | | Support for prosecutions | | | Support for reparations | | | | | |
| --- | --- | --- | --- | --- | --- | --- | --- | --- | --- | --- | --- | --- | --- | --- | --- | --- |
|  | | B (SE) | 95 % CI | | | | Beta | B (SE) | 95 % CI | Beta | B (SE) | 95 % CI | | | Beta | |
| *V1: Trapped by violence* | |  |  | | | |  |  |  |  |  |  | | |  | |
| Perceived communal empathy | | .100** (.032) | .036, .163 | | | | .156 | .082* (.035) | .013, .151 | .115 | .074** (.022) | .031, .117 | | | .172 | |
| Personal empathy | | .005 (.044) | -.082, .092 | | | | .006 | .048 (.048) | -.046, .142 | .044 | .088** (.030) | .029, .147 | | | .136 | |
| Model fit | | Adjusted R-square=.185, F(7/503)=17.519, p<.001 | | | | | | Adjusted R-square=.214, F(7/514)=21.215, p<.001 | | | Adjusted R-square=.122, F(7/537)=11.792, p<..001 | | | | | |
| *V2: Boundaries disrupting normal life* | | | | |  |  | |  |  |  |  | | |  |  | |
| Perceived communal empathy | .150*** (.032) | | | .088, .213 | | | .221 | .086* (.035) | .017, .154 | .113 | .051* (.022) | | .008, .093 | | | .111 |
| Personal empathy | .088 (.048) | | | -.005, .182 | | | .079 | .123* (.052) | .022, .225 | .100 | .174*** (.032) | | .111, .237 | | | .235 |
| Model fit | | Adjusted R-square=.220, F(7/510)=21.868, p<.001 | | | | | | Adjusted R-square=.231, F(7/524)=23.841, p<.001 | | | Adjusted R-square=.144, F(7/547)=,14.348 p<.001 | | | | | |
| *V3: Daring to resist* | |  | | |  |  | |  |  |  |  | | |  |  | |
| Perceived communal empathy | .189*** (.031) | | | .129, .249 | | | .288 | .114** (.034) | .048, .180 | .155 | .087*** (.021) | | .047, .128 | | | .198 |
| Personal empathy | -.034 (.045) | | | -.122, .055 | | | -.032 | -.004 (.050) | -.101, .094 | -.003 | .127*** (.030) | | .067, .187 | | | .183 |
| Model fit | | Adjusted R-square=.229, F(7/508)=22.857, p<.001 | | | | | | Adjusted R-square=.221, F(7/521)=22.339, p<.001 | | | Adjusted R-square=.154, F(7/544)=15.344, p<.001 | | | | | |
| *V4: Sticking together to survive* | | | | |  |  | |  |  |  |  | | |  |  | |
| Perceived communal empathy | .138*** (.032) | | | .075, .202 | | | .213 | .095** (.034) | .027, .162 | .132 | .087*** (.022) | | .044, .129 | | | .200 |
| Personal empathy | -.012 (.044) | | | -.099, .075 | | | -.013 | .090 (.047) | -.003, .183 | .086 | .096** (.029) | | .038, .153 | | | .152 |
| Model fit | | Adjusted R-square=.197, F(7/500)=18.779, p<.001 | | | | | | Adjusted R-square=.225, F(7/514)=22.627, p<.001 | | | Adjusted R-square=.135, F(7/537)=13.092, p<.001 | | | | | |

*Note.* *p<.05, **p<.01, ***p<.001. Models controlling for the same variables as in Table 2 in the main manuscript.

Table S2. Continued.

|  | | Support for truth commissions | | | | | Support for prosecutions | | | | | | | Support for reparations | | | | | | |
| --- | --- | --- | --- | --- | --- | --- | --- | --- | --- | --- | --- | --- | --- | --- | --- | --- | --- | --- | --- | --- |
|  | | B (SE) | 95 % CI | | Beta | | B (SE) | 95 % CI | | Beta | | | | B (SE) | 95 % CI | | | Beta | | |
| *V5: Friend or foe?* | |  |  | |  | |  |  | |  | | | |  |  | | |  | | |
| Perceived communal empathy | .157*** (.034) | | | .091, .223 | | .275 | .096** (.036) | .024, .167 | | | .149 | | .043 (.023) | | -.002, .087 | | | | .112 | |
| Personal empathy | -.092** (.037) | | | -.166, -.019 | | -.128 | -.013 (.041) | -.092, .067 | | | -.016 | | .053* (.025) | | .003, .103 | | | | .111 | |
| Model fit | | Adjusted R-square=.202, F(7/498)=, p<.001 | | | | | Adjusted R-square=.215, F(7/510)=21.223, p<.001 | | | | | | | Adjusted R-square=.096, F(7/533)=9.235, p<.001 | | | | | | |
| *V6: Support across frontlines* | | |  | |  | |  |  | |  | | | |  |  | | |  | | |
| Perceived communal empathy | | .085** (.032) | | .022, .147 | | .152 | .133*** (.034) | | .066, .200 | | | .213 | | .060** (.021) | .018, .101 | | | | | .162 |
| Personal empathy | | -.052 (.037) | | -.125, .021 | | -.072 | -.020 (.040) | | -.098, .058 | | | -.025 | | -.021 (.025) | -.070, .027 | | | | | -.044 |
| Model fit | | Adjusted R-square=.171, F(7/491)=15.714, p<.001 | | | | | Adjusted R-square=.223, F(7/504)=21.947, p<.001 | | | | | | | Adjusted R-square=.081, F(7/527)=7.697, p<.001 | | | | | | |
| *V7: Not in my name* | |  | |  |  | |  |  | |  | | | |  | | |  |  | | |
| Perceived communal empathy | | .099** (.036) | | .029, .169 | | .131 | .058 (.039) | -.019, .134 | | | .069 | | -.013 (.024) | | | -.060, .035 | | | | -.025 |
| Personal empathy | | .109* (.044) | | .021, .196 | | .107 | .105* (.049) | .009, .201 | | | .091 | | .146*** (.030) | | | .087, .205 | | | | .215 |
| Model fit | | Adjusted R-square=.191, F(7/502)=18.219, p<.001 | | | | | Adjusted R-square=.211, F(7/516)=20.996, p<.001 | | | | | | | Adjusted R-square=.103, F(7/538)=9.988, p<.001 | | | | | | |

*Note.* *p<.05, **p<.01, ***p<.001. Models controlling for the same variables as in Table 2 in the main manuscript.

Table S3. Linear regression models of the impact of personal and perceived communal empathy on support for transitional justice mechanisms, while controlling for diversified conflict knowledge and perceived communal prevalence.

|  | Support for truth commissions | | | Support for prosecutions | | | Support for reparations | | |
| --- | --- | --- | --- | --- | --- | --- | --- | --- | --- |
|  | B (SE) | 95 % CI | Beta | B (SE) | 95 % CI | Beta | B (SE) | 95 % CI | Beta |
| Perceived communal empathy | .227*** (.042) | .144, .310 | .309 | .147** (.046) | .057, .237 | .178 | .059* (.027) | .006, .113 | .118 |
| Personal empathy | .009 (.065) | -.118, .136 | .007 | .117 (.071) | -.023, .257 | .087 | .233*** (.042) | .150, .315 | .287 |
| Diversified conflict knowledge | -.043* (.019) | -.081, -.006 | -.093 | -.020 (.021) | -.061, .022 | -.038 | -.102*** (.012) | -.126, -.078 | -.328 |
| Perceived communal prevalence | -.063 (.037) | -.136, .010 | -.132 | -.059 (.041) | -.140, .022 | -.110 | .012 (.024) | -.035, .059 | .038 |
| Model fit | Adjusted R-square=.221, F(9/496)=16.879, p<.001 | | | Adjusted R-square=.229, F(9/510)=18.155, p<.001 | | | Adjusted R-square=.237, F(9/533)=19.702, p<..001 | | |

*Note.* *p<.05, **p<.01, ***p<.001. Models controlling for the same variables as in Table 2 in the main manuscript.

Table S3 presents regression analyses while controlling for *Diversified conflict knowledge* and *Perceived communal prevalence.*

After each vignette, respondents were asked whether they know about a similar event: specifically, they were asked whether they have personally witnessed it, heard it from an acquaintance who personally witnessed it, or learned about it through media. *Diversified conflict knowledge* was operationalized a number of vignettes known through any of the three sources, and ranges from 0 to 7.

*Perceived communal prevalence* was assessed with the item “Thinking about the period of conflict, how widespread were events like this one: In the community where you lived during the conflict”, rated on a 1 (Very rare) to 6 (Very common) scale after each vignette. The composite score was computed as the mean across 7 items (Cronbach’s alpha is .96).
